# Heterogeneity of eHealth literacy and treatment burden in older adults with heart failure: a multidimensional latent profile analysis

**DOI:** 10.3389/fpubh.2026.1822855

**Published:** 2026-06-02

**Authors:** Xiaoxia Wu, Yuejun Wang, Deyan Gao, Xialing Dai

**Affiliations:** 1 Department of Cardiology, Tongde Hospital of Zhejiang Province Affiliated to Zhejiang Chinese Medical University (College of Integrated Traditional Chinese and Western Medicine Clinical Medicine), Hangzhou, Zhejiang, China; 2 Department of Geriatrics, Jinhua Road Campus, The Affiliated Hospital of Hangzhou Normal University (Zhejiang Geriatric Care Hospital), Hangzhou, Zhejiang, China

**Keywords:** eHealth literacy, heart failure, latent profile analysis, older adults, person-centered care, treatment burden

## Abstract

**Background:**

Heart failure (HF) management imposes a substantial multidimensional treatment burden (BoT) on older adults. While digital health interventions offer potential solutions, their success heavily depends on patients' eHealth literacy (eHL). Previous variable-centered research evaluating total scale scores has systematically masked the individual heterogeneity and dimensional interplay between eHL and BoT.

**Objective:**

To identify the multidimensional latent profiles of eHealth literacy and treatment burden among older adults with chronic HF, and to explore the independent sociodemographic and clinical predictors of these profiles.

**Methods:**

A cross-sectional study was conducted involving 425 older adults with HF in China. Data were collected using demographic/clinical abstraction forms, the Chinese eHealth Literacy Scale (C-eHEALS), and the Patient Experience with Treatment and Self-Management (PETS). Latent profile analysis (LPA) was executed using 14 specific continuous dimensional indicators from these scales. Multinomial logistic regression was utilized to identify factors influencing profile membership.

**Results:**

Three distinct latent profiles were identified. As visually supported by the standardized Z-score patterns, the “Vulnerable” profile (28.9%) exhibited a starkly inverse clinical state characterized by profound deficits across all eHL dimensions and overwhelmingly high systemic BoT. The “Capable” profile (22.1%) demonstrated the opposite, optimal pattern (high eHL, minimal BoT). The “Transitional” profile (48.9%) displayed intermediate scores but high dimensional discordance (e.g., adequate information acquisition but poor evaluation, paired with high diet/exercise burdens). Multinomial logistic regression revealed that older age, primary-level education, living alone, and a higher Charlson Comorbidity Index were significant independent predictors of membership in the Vulnerable profile.

**Conclusion:**

Older adults with HF do not experience digital health demands and self-care workloads uniformly. Identifying these highly distinct, dimension-specific typologies highlights the potential value of moving away from “one-size-fits-all” digital deployments toward precision-based, stratified care models designed to mitigate specific vulnerabilities.

## 1 Introduction

Heart failure (HF) represents a profound and escalating global public health crisis, characterized by a complex clinical trajectory, high readmission rates, and substantial multidimensional morbidity, particularly among the older adult population (1, 2). This crisis is particularly acute in China, which is currently experiencing an unprecedented acceleration in population aging. By the end of 2024, China's population aged 60 and above had reached 310.31 million, accounting for 22% of the total population (3). Concurrently, the epidemiological burden of cardiovascular diseases has surged, with the prevalence and incidence of HF escalating substantially alongside this demographic shift (4). While the rapid nationwide integration of digital health technologies offers promising avenues for managing this chronic disease burden, it simultaneously exposes Chinese older adults to a widening “digital divide.” Many older patients face severe realistic dilemmas, ranging from structural barriers in internet access to suboptimal digital health literacy and technology anxiety, which systematically hinder their ability to utilize emerging eHealth resources effectively (5). The contemporary management of HF necessitates rigorous self-care behaviors, adherence to intricate polypharmacy regimens, dietary restrictions, and continuous vigilant symptom monitoring, all of which impose a heavy and enduring treatment burden (BoT) on patients (6).

Treatment burden is conceptualized as the cumulative workload of healthcare regimens and the subsequent detrimental impact of this workload on patient functioning, psychological wellbeing, and health-related quality of life (6). Concurrently, the rapid proliferation of digital health interventions—encompassing telemonitoring, patient portals, and mobile health applications—has offered transformative potential for mitigating HF exacerbations and enhancing self-care efficacy (7). However, the successful utilization of these digital modalities is heavily contingent upon a patient's eHealth literacy (eHL), defined as the cognitive and social capacity to seek, understand, appraise, and apply electronic health information to solve health problems (8, 9). Older adults with HF frequently exhibit limited eHL, which paradoxically exacerbates their treatment burden by introducing novel technological stressors and navigational barriers, thereby undermining self-care efficacy and accelerating clinical decline (10, 11).

Despite the growing theoretical recognition of the intricate interplay between eHL and treatment burden, the existing cardiovascular nursing literature has predominantly relied on variable-centered analytical methodologies, focusing almost exclusively on aggregated total scale scores (12, 13). While these conventional approaches successfully elucidate the global inverse relationships between health literacy and disease burden, they operate on the fundamental assumption of population homogeneity and systematically mask the critical dimensional heterogeneity inherent within the older adult HF population (12). Clinical reality demonstrates that patients possess highly distinct, idiosyncratic configurations of technological adaptability and perceived healthcare workload (13). For instance, relying on total scores fails to differentiate a patient who excels at finding online health information but lacks the critical skills to evaluate its credibility, from a patient who entirely lacks internet access (1, 2). Similarly, a patient might experience overwhelming burden specifically regarding daily dietary restrictions and exercise, while reporting minimal burden concerning medical expenses or healthcare system navigation.

Identifying these granular, dimensional typologies is essential for operationalizing the Situation-Specific Theory of Heart Failure Self-Care, which posits that highly specific patient characteristics and environmental complexities directly govern self-care maintenance and management decisions (14). A person-centered analytical approach, specifically Latent Profile Analysis (LPA), addresses this critical methodological gap by classifying individuals into mutually exclusive, unobserved latent subgroups based on their continuous response patterns across multiple specific dimensions, thereby enabling the development of highly targeted, profile-specific nursing interventions (15–17).

Therefore, the overarching objective of this study is to utilize Latent Profile Analysis to explore the unobserved heterogeneity and characterize the latent profiles of eHealth literacy and treatment burden based on their specific dimensional sub-components among older adults with HF. Specifically, the study aims to: (1) empirically identify distinct latent profiles based on 14 specific dimensional indicators of eHL and BoT; (2) rigorously examine the baseline demographic, clinical, and psychosocial differences across these identified profiles; and (3) determine the independent predictive factors that are significantly associated with profile membership. By delineating these multidimensional latent typologies, this research seeks to provide cardiovascular nursing educators and clinicians with an evidence-based framework to deliver precision-based digital health interventions and proactively mitigate specific facets of treatment burden.

## 2 Methods

### 2.1 Study design and ethical declarations

This study employed a descriptive, exploratory, cross-sectional design to systematically investigate the latent dimensional profiles of eHealth literacy and treatment burden within a cohort of older adults diagnosed with heart failure. The methodological framework, data collection procedures, and manuscript reporting adhered strictly to the Strengthening the Reporting of Observational Studies in Epidemiology (STROBE) guidelines for cross-sectional studies to ensure methodological rigor and transparency (18). The research protocol underwent comprehensive ethical review and was formally approved by the Zhejiang Geriatric Care Hospital (Approval Number: 2025–001). The study was conducted in full compliance with the ethical principles outlined in the Declaration of Helsinki regarding medical research involving human subjects. Prior to the initiation of any data collection procedures, all eligible participants were provided with detailed, plain-language information regarding the study's purpose, the voluntary nature of their participation, the absence of any impact on their standard clinical care, and the strict confidentiality protocols governing their personal health information. Informed consent was obtained from all participants. For older adults who exhibited age-related visual impairments or literacy limitations, trained cardiovascular research nurses read the informed consent documents aloud and obtained documented verbal consent in the presence of an impartial witness, a specific alternative procedure explicitly approved by the ethics committee, thereby strictly aligning with established nursing research ethical standards for vulnerable geriatric populations.

### 2.2 Participants and setting

Participants were prospectively recruited using a convenience sampling methodology from the cardiovascular outpatient clinics and inpatient wards of Zhejiang Geriatric Care Hospital in mainland China between September 2025 and early March 2026. To ensure a high degree of clinical relevance and to control for confounding variables, the inclusion criteria were rigorously established: (1) chronological age of 65 years or older; (2) a confirmed primary clinical diagnosis of chronic heart failure—inclusive of heart failure with reduced ejection fraction (HFrEF), mildly reduced ejection fraction (HFmrEF), or preserved ejection fraction (HFpEF)—documented in the electronic medical record for a minimum of 6 months prior to enrollment; (3) current classification of New York Heart Association (NYHA) functional class I, II, III, or IV; (4) stable hemodynamic status at the time of assessment; and (5) basic ownership of, or consistent access to, an internet-enabled digital device. According to the 57th Statistical Report on China's Internet Development (February 2026), the mobile internet penetration rate among Chinese adults aged 65 and older is 50% (19). Including the remaining unconnected population would inevitably result in a severe floor effect on the C-eHEALS scale. Therefore, this criterion was deliberately established to isolate the “second-level digital divideis criterion was deliberately established to isolate the “evere floor effect on the C-eHEALSnternet-enabled digital device. According) (21, 22). This ensures the latent profile analysis accurately captures multidimensional cognitive heterogeneity among those who actively engage with digital health platforms.

The exclusion criteria were carefully delineated to preserve data integrity: (1) severe cognitive impairment, operationalized as a Mini-Mental State Examination (MMSE) score of less than 24; (2) active, severe psychiatric disorders documented in the medical record; (3) profound visual, auditory, or motor deficits that would physically prevent the use of digital devices; and (4) concurrent participation in another structured eHealth, telemonitoring, or self-care clinical trial within the preceding 3 months.

The determination of an appropriate sample size was guided by the statistical requirements specific to dimensional Latent Profile Analysis. Methodological simulation studies and established guidelines for Gaussian mixture modeling stipulate that parameter estimation requires a minimum of 300 to 500 cases to ensure the stability of the maximum likelihood estimates and adequate statistical power for the Bootstrap Likelihood Ratio Test (BLRT) (15, 16). Assuming a conservative 15% attrition or missing data rate due to the older age of the cohort, a total of 500 survey packets were distributed. Following rigorous data screening, 425 fully valid responses were retained for the final analysis.

### 2.3 Measures and indicators

Data collection was executed utilizing a comprehensive, structured survey packet comprising two extensively validated Chinese-version psychometric instruments and a standardized demographic and clinical data abstraction form.

#### 2.3.1 Demographic and clinical characteristics

A structured abstraction form was utilized to collect self-reported demographic data, encompassing chronological age, biological sex, highest level of educational attainment, marital status, monthly household income, and living arrangements. Core clinical variables were extracted directly from the hospital's electronic medical records by the clinical research team, including NYHA functional class, left ventricular ejection fraction (LVEF), disease duration, and the total number of daily prescribed cardiovascular medications. Furthermore, the age-adjusted Charlson Comorbidity Index (CCI) was utilized to rigorously quantify the cumulative burden of comorbid conditions, providing a vital metric of objective physiological burden (20).

#### 2.3.2 eHealth literacy scale (C-eHEALS)

Given the study setting in mainland China, we utilized the validated simplified Chinese version of the eHEALS (C-eHEALS) (21). The C-eHEALS is an 8-item psychometric instrument designed to evaluate patients' perceived capacity to navigate electronic health information. To capture the precise dimensional heterogeneity required for LPA, the 8 items were mapped onto three core underlying dimensions: Information Acquisition (Items 1–3), Interactive Evaluation (Items 4–7), and Decision-Making Application (Item 8). Items are scored on a 5-point Likert scale ranging from 1 (“strongly disagree”) to 5 (“strongly agree”). To ensure standardization across dimensions with varying numbers of items, the domain mean score (range 1–5) for each of the three dimensions was calculated and utilized as an independent indicator in the LPA model. In the current study sample, the instrument exhibited exceptional overall reliability (Cronbach's alpha = 0.92; see Supplementary Table 1).

#### 2.3.3 Patient experience with treatment and self-management (PETS)

The subjective workload and distress associated with managing HF were quantified using the translated Chinese short-form Patient Experience with Treatment and Self-Management (PETS), which has been rigorously validated in Chinese patients with multimorbidity (22). This instrument comprises 32 items strictly categorized into 11 distinct dimensions: Medical Information (6 items), Medication (2 items), Medical Appointments (2 items), Health Management (2 items), Medication Side Effects (1 item), Diet (2 items), Exercise (2 items), Medical Expenses (4 items), Healthcare System (2 items), Social Roles (4 items), and Physical/Mental Exhaustion (5 items). All items measure the difficulty of executing self-management tasks on a 5-point Likert scale ranging from 1 (“no difficulty”) to 5 (“completely unable to execute”). As explicitly recommended by the scale's official scoring guidelines to eliminate weighting biases introduced by the varying number of items across domains and to optimize LPA model fitting, the domain mean score (range 1–5) for each of the 11 dimensions was calculated and utilized as independent indicators. The overall Cronbach's alpha for the PETS in this sample was 0.882.

### 2.4 Statistical analysis

Data preparation, comprehensive descriptive statistics, and inferential analyses were conducted using IBM SPSS Statistics (Version 26.0), while the core Latent Profile Analysis was executed using Mplus (Version 8.3). The analytical workflow proceeded through sequential phases:

First, descriptive statistics were generated to summarize the cohort. The distributions of the eHEALS and PETS scores were evaluated for normality using the Kolmogorov-Smirnov test prior to subsequent advanced modeling (Supplementary Figure 1). Second, LPA, a sophisticated person-centered Gaussian mixture modeling technique, was utilized to identify the unobserved subpopulations. To accurately capture individual heterogeneity, the model utilized 14 continuous manifest indicators: the 3 standardized domain mean scores of the C-eHEALS and the 11 standardized domain mean scores of the PETS. Models specifying 1 to 5 latent classes were sequentially estimated using the Robust Maximum Likelihood (MLR) estimator (15, 16). Model fit and the final enumeration of classes were determined using a strict convergence of multiple statistical indices: the Akaike Information Criterion (AIC), Bayesian Information Criterion (BIC), sample-size adjusted BIC (aBIC), Entropy (>0.80), the Lo-Mendell-Rubin adjusted Likelihood Ratio Test (LMR-LRT, *P* < 0.05), and the Bootstrap Likelihood Ratio Test (BLRT, *P* < 0.05).

Third, following the establishment of the optimal latent profile model, each participant was deterministically assigned to the specific profile for which they possessed the highest posterior probability. To evaluate the baseline differences across the identified profiles, one-way Analysis of Variance (ANOVA) and Pearson's Chi-square tests were employed. Finally, a multinomial logistic regression model was constructed to identify the independent predictors of latent profile membership, calculating Odds Ratios (OR) and 95% Confidence Intervals (CI).

Third, to evaluate the baseline differences across the identified profiles, one-way Analysis of Variance (ANOVA) and Pearson's Chi-square tests were employed. Finally, to rigorously identify the independent predictors of latent profile membership while accounting for the classification uncertainty inherent in LPA, we executed the advanced bias-adjusted three-step approach (R3STEP procedure) directly within the Mplus environment, as outlined by Asparouhov and Muthén (23). This robust method utilizes the inverse logits of classification error rates as weights, yielding bias-adjusted Odds Ratios (OR) and 95% Confidence Intervals (CI).

**Figure 1:**
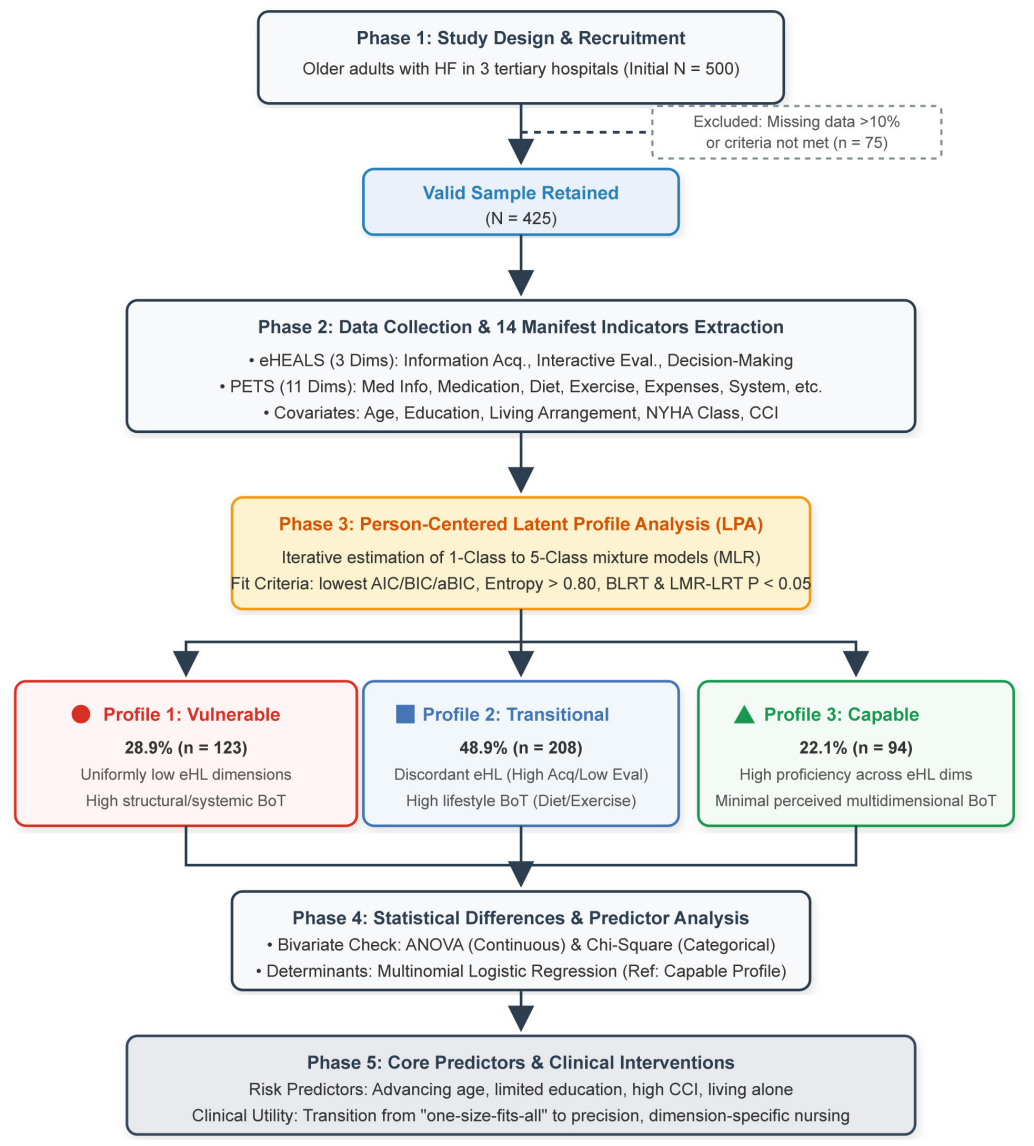
Flowchart of the study design, participant recruitment, and Latent Profile Analysis (LPA) methodology.

Furthermore, a rigorous sensitivity analysis was conducted by excluding all asymptomatic patients (NYHA Class I) and re-estimating the LPA models to verify that the identified latent profile structure was not artificially driven by patients with minimal clinical symptoms. The comprehensive workflow encompassing participant recruitment, data screening, and the sequential execution of the Latent Profile Analysis (LPA) is systematically illustrated in Figure 1.

### 2.5 Quality control

Rigorous quality control protocols were implemented throughout the entire study lifecycle. All clinical data collectors were registered nurses holding at least a Bachelor of Science in Nursing (BSN) degree and underwent standardized training on research ethics and non-directive clinical interviewing techniques. Completed questionnaires were audited weekly. Any survey exhibiting more than 10% missing data across the primary measurement scales was categorically discarded. Due to rigorous on-site quality control by trained nurses, the completeness of the retrieved surveys was highly polarized; consequently, no collected questionnaires in our final dataset exhibited a missing data rate between 5% and 10%. For completely random missing data falling below the stringent 5% threshold, Full Information Maximum Likelihood (FIML) estimation was seamlessly utilized within the Mplus environment during the LPA phase, which directly estimates parameters using all available data, thereby preserving statistical power and avoiding attenuation biases (16).

## 3 Results

### 3.1 Baseline demographic and clinical characteristics

Following the application of the rigorous inclusion and exclusion criteria, a total of 425 older adults with heart failure were included in the final analysis. The comprehensive demographic and clinical profiles of the cohort characterize a specific, highly multi-morbid geriatric HF population encountered in this local tertiary cardiovascular care setting. The mean chronological age of the participants was 74.2 years (SD = 6.8), with a slight male predominance (55.3%, *n* = 235). Reflecting broader generational trends in the region, a significant proportion of the cohort exhibited limited educational attainment, with nearly half of the participants (48.0%, *n* = 204) having completed only primary education or lower. Regarding living arrangements, 23.1% (*n* = 98) of the older adults reported living alone, a critical factor related to social support and caregiver contribution.

Clinically, the cohort demonstrated substantial disease burden. The majority of patients were classified as having NYHA functional class II (44.7%, *n* = 190) or class III (34.1%, *n* = 145) symptoms, indicating moderate to severe physical limitations in daily activities, while the remaining participants were equally distributed between NYHA Class I (10.6%, *n* = 45) and Class IV (10.6%, *n* = 45). The mean duration since the initial HF diagnosis was 4.6 years (SD = 3.2). The overarching burden of systemic comorbidity was notably high, as evidenced by an average age-adjusted Charlson Comorbidity Index (CCI) score of 2.8 (SD = 1.4). Furthermore, 43.5% of the sample scored ≥ 3 on the CCI, indicating a profound and complex systemic disease burden that complicates isolated HF management15. Polypharmacy was universally prevalent, with participants managing an average of 7.2 (SD = 2.5) prescribed medications daily. Detailed baseline characteristics for the overall sample are systematically summarized in Table 1.

**Table 1:** Baseline demographic and clinical characteristics of the study sample (*N* = 425).

Variables	Total (*N* = 425)
Age (years), Mean (SD)	74.2 (6.8)
**Sex**, ***n*** **(%)**	
Male	235 (55.3)
Female	190 (44.7)
**Education level**, ***n*** **(%)**	
Primary or below	204 (48.0)
Secondary	158 (37.2)
Tertiary or above	63 (14.8)
**Living arrangement**, ***n*** **(%)**	
Living alone	98 (23.1)
Living with family	327 (76.9)
**NYHA class**, ***n*** **(%)**	
Class I	45 (10.6)
Class II	190 (44.7)
Class III	145 (34.1)
Class IV	45 (10.6)
Charlson comorbidity index, Mean (SD)	2.8 (1.4)
Daily medications (*n*), Mean (SD)	7.2 (2.5)

### 3.2 Latent profile model fit and class enumeration

To empirically identify the unobserved subpopulation structure based on the nuanced convergence of eHealth literacy and treatment burden, LPA was executed using the 14 dimensional mean scores. The comprehensive model fit indices are presented in Table 2. As the number of specified latent classes increased sequentially from 1 to 3, the absolute information criteria (AIC, BIC, aBIC) demonstrated a continuous and substantial decrease (Supplementary Figure 2), indicating a significant improvement in overall model fit. The 3-class model yielded an excellent Entropy value of 0.884, indicating a high degree of classification precision. Furthermore, both the LMR-LRT (*P* = 0.012) and the BLRT (*P* \ < 0.001) achieved statistical significance for the 3-class solution, statistically confirming its superiority over the 2-class model. When evaluating the 4-class model, the LMR-LRT became statistically non-significant (*P* = 0.285), and it produced a spurious class containing merely 4.2% of the sample, violating statistical parsimony. Consequently, the 3-class model was selected as the optimal fit.

**Table 2:** Model fit indices for latent profile analysis of ehealth literacy and treatment burden (*N* = 425).

Model	AIC	BIC	aBIC	Entropy	LMR-LRT (*P*-value)	BLRT (*P*-value)	Class proportions (%)
1–Class	8452.14	8516.98	8464.32	–	–	–	100
2–Class	7824.56	7917.80	7842.06	0.852	< 0.001	< 0.001	38.6/61.4
**3–Class**	**7410.22**	**7531.84**	**7433.05**	**0.884**	**0.012**	**< 0.001**	**28.9/48.9/22.1**
4–Class	7385.41	7535.43	7413.58	0.865	0.285	< 0.001	4.2/26.5/48.0/21.3
5–Class	7350.18	7528.60	7383.69	0.871	0.410	0.065	3.8/15.2/20.4/41.1/19.5

AIC, Akaike Information Criterion; BIC, Bayesian Information Criterion; aBIC, sample-size adjusted BIC; LMR-LRT, Lo-Mendell-Rubin adjusted Likelihood Ratio Test; BLRT, Bootstrap Likelihood Ratio Test. Bold formatting indicates the selected optimal model. Bold values indicate the selected optimal latent profile model.

Based on the distinct dimensional scoring patterns, the latent profiles were named:

1. **Profile 1** (28.9%, *n* = 123): Characterized by profound deficits across all eHL dimensions and overwhelmingly high burdens across all structural and systemic PETS dimensions. Designated as the “Vulnerable” profile.
2. **Profile 2** (48.9%, *n* = 208): Exhibited significant dimensional discordance—capable of acquiring info but poor at evaluating it, leading to highly specific burdens in lifestyle and behavioral management. Designated as the “Transitional” profile.
3. **Profile 3** (22.1%, *n* = 94): Characterized by high proficiency across all eHL dimensions and minimal perceived BoT across all domains. Designated as the “Capable” profile.

To address potential clinical heterogeneity and ensure these profiles were not artificially driven by asymptomatic individuals, we conducted a sensitivity analysis excluding all 45 NYHA Class I patients (remaining *n* = 380). As detailed in Supplementary Table 3, the 3-class solution remained robustly the optimal fit (Entropy = 0.870, LMR-LRT *P* = 0.015, BLRT *P* < 0.001). The emergent class proportions remained highly stable (31.1% Vulnerable, 50.0% Transitional, 18.9% Capable), confirming that the “Capable” profile represents a genuine cognitive-behavioral phenotype rather than a mere artifact of minimal physical symptoms.

### 3.3 Core indicator characteristics across latent profiles

**Figure 2:**
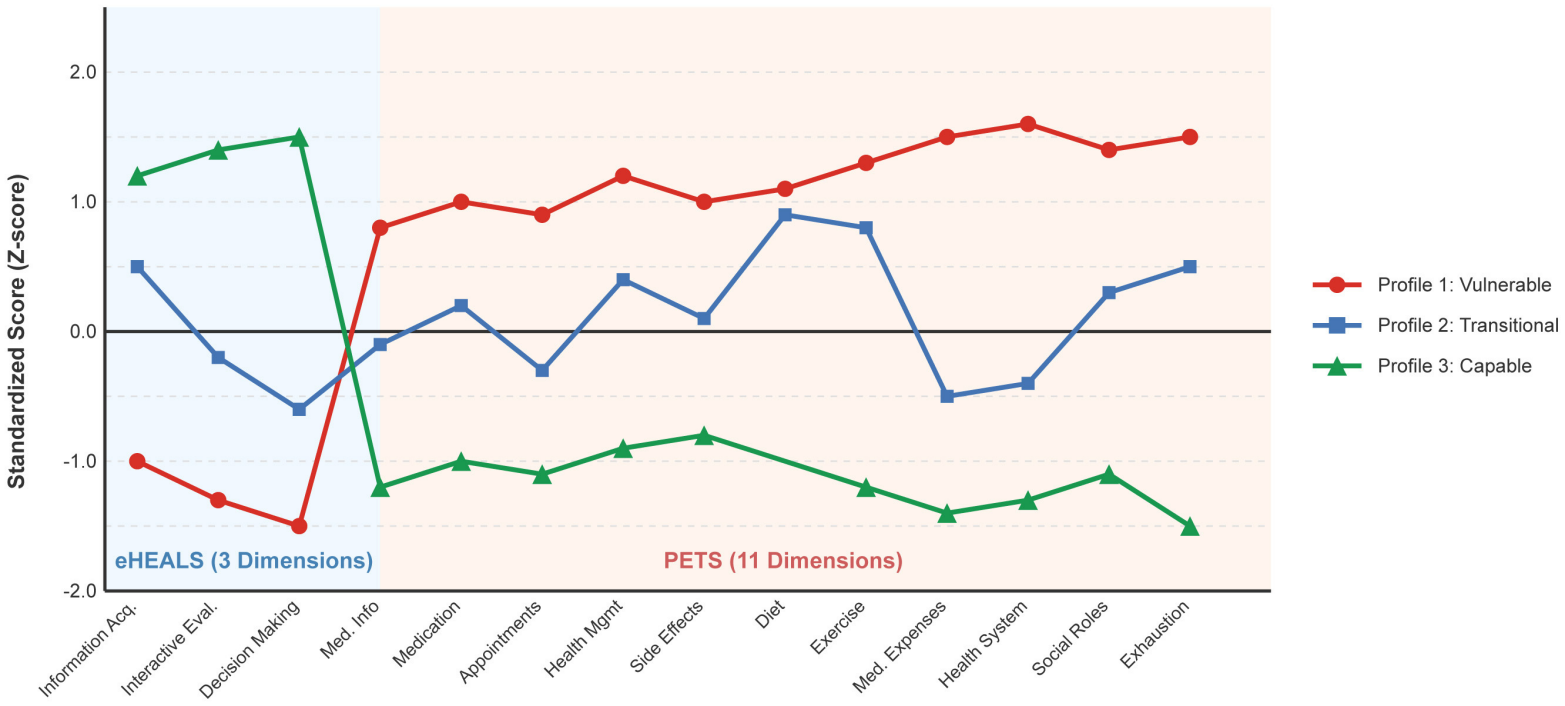
Standardized Dimensional Scores of eHealth Literacy and Treatment Burden by Latent Profile. eHL, eHealth Literacy; eHEALS, eHealth Literacy Scale; PETS, Patient Experience with Treatment and Self-Management; Z-score, Standardized Score. *Description:* This line graph illustrates the standardized mean scores (Z-scores) across 14 specific dimensions (3 from eHEALS and 11 from PETS) for the three identified latent profiles. The Y-axis represents the Z-score, where 0.0 is the overall sample mean. The X-axis displays the specific dimensions. The red line (Profile 1: Vulnerable) shows uniformly low eHealth literacy and extremely high treatment burden, particularly in structural dimensions. The blue line (Profile 2: Transitional) demonstrates a discordant pattern with adequate information acquisition but poor evaluation skills, coupled with high lifestyle-related burdens (diet and exercise). The green line (Profile 3: Capable) indicates high eHealth literacy across all dimensions and minimal treatment burden.

To facilitate clinical interpretation of the dimensional heterogeneity, the standardized dimensional means (Z-scores) for the 14 indicators across the three latent profiles are vividly depicted in Figure 2.

As explicitly demonstrated in the dimensional breakdown of Figure 1, identifying profiles by individual dimensions reveals crucial clinical nuances that aggregate scores conceal. The “Vulnerable” profile (Profile 1) exhibited starkly depressed scores across all three eHL dimensions, coupled with overwhelmingly high burdens that peaked structurally in the Healthcare System (Z≈+1.6), Medical Expenses (Z≈+1.5), and Physical/Mental Exhaustion (Z≈+1.5) dimensions.

Crucially, the dimensional analysis exposed a highly discordant pattern in the “Transitional” profile (Profile 2). These patients demonstrated above-average capabilities in Information Acquisition (Z≈+0.5) but scored profoundly poorly in Interactive Evaluation (Z≈-0.2) and Decision-Making (Z≈-0.6). Correspondingly, their treatment burden was not systemic but highly localized to lifestyle execution, showing sharp spikes in the Diet (Z≈+0.9) and Exercise (Z≈+0.8) dimensions. Conversely, the “Capable” profile (Profile 3) demonstrated high proficiency across all literacy dimensions, protecting them from substantial burden across all PETS metrics.

### 3.4 Baseline differences across profiles

Bivariate analyses revealed profound and statistically significant differences in baseline demographic and clinical characteristics across the three distinct latent profiles, as systematically detailed in Table 3. The Analysis of Variance (ANOVA) and Chi-square tests indicated that patients classified within the “Vulnerable” profile were significantly older (Mean = 77.5 vs. 70.8 years, *F* = 26.41, *P* < 0.001), possessed a drastically higher prevalence of only primary-level education (68.3%, χ^2^ = 48.62, *P* < 0.001), and were substantially more likely to live alone without immediate familial support (34.1%) when compared directly to the “Capable” profile.

**Table 3:** Bivariate analysis of baseline demographic and clinical characteristics across latent profiles.

Variables	Profile 1: Vulnerable (*n* = 123)	Profile 2: Transitional (*n* = 208)	Profile 3: Capable (*n* = 94)	Statistic (F/χ^2^)	*P*–value
Age (years), Mean (SD)	77.5 (6.2)	73.8 (6.5)	70.8 (5.7)	26.41	< 0.001
**Sex**, ***n*** **(%)**					
Male	62 (50.4)	118 (56.7)	55 (58.5)	3.25	0.197
Female	61 (49.6)	90 (43.3)	39 (41.5)		
**Education level**, ***n*** **(%)**					
Primary or below	84 (68.3)	100 (48.1)	20 (21.3)	48.62	< 0.001
Secondary	32 (26.0)	80 (38.5)	46 (48.9)		
Tertiary or above	7 (5.7)	28 (13.5)	28 (29.8)		
**Living arrangement**, ***n*** **(%)**					
Living alone	42 (34.1)	44 (21.2)	12 (12.8)	14.38	0.006
Living with family	81 (65.9)	164 (78.8)	82 (87.2)		
**NYHA class**, ***n*** **(%)**					
Class I	5 (4.1)	18 (8.7)	22 (23.4)	38.54	< 0.001
Class II	38 (30.9)	102 (49.0)	50 (53.2)		
Class III	60 (48.8)	65 (31.3)	20 (21.3)		
Class IV	20 (16.3)	23 (11.1)	2 (2.1)		
Charlson comorbidity index, Mean (SD)	3.6 (1.5)	2.6 (1.2)	2.0 (1.1)	41.22	< 0.001
Daily medications (*n*), Mean (SD)	8.8 (2.6)	6.8 (2.2)	5.9 (1.9)	45.70	< 0.001

Clinically, the “Vulnerable” group experienced a significantly heavier physiological disease burden. This was characterized by a preponderance of advanced NYHA functional classes (Class III/IV = 65.1%), elevated Charlson Comorbidity Indices (*F* = 41.22, *P* < 0.001), and a much heavier daily polypharmacy regimen (Mean = 8.8 medications). In stark contrast, the “Capable” profile comprised the youngest older adults in the cohort, characterized by higher educational attainment, fewer compounding comorbid conditions, and predominantly mild (NYHA Class I/II) physical symptoms.

### 3.5 Multinomial logistic regression analysis of influencing factors

To meticulously control for confounding variables and to identify the independent, robust determinants of latent profile membership, a bias-adjusted three-step multinomial logistic regression model was executed using the significant variables identified in the prior univariate analysis (Supplementary Table 2). In this model, the “Capable” profile (Profile 3) intentionally served as the baseline reference group.

As detailed exhaustively in Table 4, advancing chronological age significantly elevated the odds of assignment to the “Vulnerable” profile (OR = 1.25, 95% CI: 1.14–1.38). Higher educational attainment served as a robust, independent protective factor. Furthermore, the objective clinical burden heavily dictated profile membership. Each one-unit increase in the Charlson Comorbidity Index incrementally increased the odds of falling into the “Vulnerable” profile by 2.88 times (95% CI: 1.85–4.45). Similarly, patients suffering from advanced NYHA classes (III/IV) had 4.50 times higher odds (95% CI: 2.15–9.45) of being trapped in the “Vulnerable” group compared to those in NYHA I/II. Finally, living alone independently increased the risk of experiencing high treatment burden and low eHL (Profile 1 vs. 3: OR = 3.65, 95% CI: 1.52–8.76), emphatically highlighting the critical role of the immediate social microsystem.

**Table 4:** Bias–adjusted three–step multinomial logistic regression analysis of factors influencing latent profile membership.

Variables	Profile 1: Vulnerable vs. Profile 3: Capable (Ref)		Profile 2: Transitional vs. Profile 3: Capable (Ref)	
	Adjusted OR (95% CI)	*P*–value	Adjusted OR (95% CI)	*P*–value
Age (per 1–year increase)	1.25 (1.14, 1.38)	< 0.001	1.09 (1.01, 1.18)	0.032
**Education level (Ref: Primary)**				
Secondary	0.29 (0.12, 0.71)	0.007	0.44 (0.22, 0.88)	0.02
Tertiary or above	0.08 (0.02, 0.35)	< 0.001	0.21 (0.08, 0.55)	0.002
**Living arrangement (Ref: With family)**				
Living alone	3.65 (1.52, 8.76)	0.004	1.85 (0.85, 4.05)	0.12
**NYHA class (Ref: Class I/II)**				
Class III/IV	4.50 (2.15, 9.45)	< 0.001	1.92 (1.02, 3.65)	0.044
Charlson comorbidity index (per 1 unit)	2.88 (1.85, 4.45)	< 0.001	1.65 (1.18, 2.30)	0.004
Daily Medications (per 1 medication)	1.30 (1.10, 1.55)	0.002	1.11 (0.96, 1.28)	0.165

OR, Odds Ratio; CI, Confidence Interval. The multinomial regression model adjusted for all listed variables simultaneously using the R3STEP bias-adjusted procedure to account for classification uncertainty.

## 4 Discussion

This comprehensive study utilized Latent Profile Analysis across 14 discrete psychometric dimensions to uncover the complex, previously unobserved heterogeneity of eHealth literacy and treatment burden among older adults living with chronic heart failure. By transitioning from traditional total-score variables to dimension-level indicators, the researchers successfully identified three highly distinct clinical typologies: the “Vulnerable” profile (28.9%), the “Transitional” profile (48.9%), and the “Capable” profile (22.1%). These empirical findings provide compelling evidence that older adults do not experience digital health demands and self-care workloads uniformly; rather, their struggles are highly dimensional and heavily stratified by intersecting demographic, clinical, and social determinants.

The identification of the ypologies: the “Vulnerable” profile (28.9%), the “Transitional” proeHL domains and staggering burdens regarding the healthcare system, expenses, and physical exhaustion–aligns well with the core tenets of Riegel's Situation-Specific Theory of Heart Failure Self-Care (14). In the context of our study, these patients epitomized profoundly compromised capacity; they were significantly older, socially isolated, and burdened by severe, overlapping comorbidities. Our dimensional findings suggest a potential association where extreme physical exhaustion (a specific PETS dimension) co-occurs with lower cognitive reserves necessary to acquire and evaluate novel eHealth platforms, which may relate to digital disenfranchisement. Consequently, this might limit their access to vital telemonitoring that could alleviate their overarching systemic workload, thereby potentially accelerating their clinical decline (2, 6, 13).

Crucially, the dimensional LPA revealed a profound discordance within the “Transitional” profile, a nuance entirely missed by previous total-score studies. These patients demonstrated adequate skills in *Information Acquisition* but critical deficiencies in *Interactive Evaluation* and *Decision-Making*. From a treatment burden perspective, they were not overwhelmed by systemic issues (like expenses or appointments), but rather by the daily execution of lifestyle modifications, notably *Diet* and *Exercise* (24). This suggests a classic “information-application gap”: while they can successfully search for HF diets online, their inability to evaluate conflicting health information leaves them frustrated and unable to confidently implement dietary changes, artificially inflating their specific lifestyle burden. Conversely, the “Capable” profile demonstrated optimal dimensional adaptation across the board, echoing cardiovascular literature that higher foundational evaluation skills serve as a potent protective buffer against the psychosocial strain of chronic disease management (7, 25).

When comparing these nuanced results with the broader literature, our multinomial regression confirmed that the Charlson Comorbidity Index and polypharmacy are aggressive, independent drivers of overall profile assignment (20). Notably, living alone emerged as a profound risk factor for assignment to the Vulnerable profile. This substantiates recent frameworks emphasizing the indispensable role of the immediate “microsystem” (1, 14). Older adults living alone acutely lack the surrogate technological operation and emotional scaffolding provided by family members, forcing them to absorb the entirety of the systemic medical workload autonomously.

### 4.1 Clinical nursing practice implications

The identification of these dimensional profiles highlights the clinical utility of moving away from monolithic educational models toward precision-based interventions.

1. **For the Vulnerable Profile:** Interventions must target the structural and exhaustion dimensions of burden. Digital tools should be bypassed entirely in favor of analog, high-touch interventions (e.g., intensive home nursing visits, tangible pillbox pre-packaging). If digital tools are utilized, they must feature “zero-interface” passive monitoring requiring absolutely no active patient input. Interdisciplinary social work consultations are imperative to combat their severe expense and systemic navigation burdens.
2. **For the Transitional Profile:** Nursing educators must move beyond teaching basic internet searches. Interventions must specifically target the *Interactive Evaluation* and *Decision-Making* dimensions of eHL through structured “teach-back” methods and critical appraisal workshops. Concurrently, nurses should directly address their *Diet* and *Exercise* burdens by translating complex online nutritional information into highly simplified, unambiguous, and culturally tailored action plans.
3. **For the Capable Profile:** Nurses can aggressively implement advanced eHealth self-management tools, such as interactive symptom-tracking applications and patient-led diuretic titration protocols, maximizing clinical outcomes and drastically reducing clinic visit burdens.

### 4.2 Limitations

This study possesses several methodological limitations. First, the cross-sectional design inherently precludes the definitive establishment of causal relationships between the evolution of eHL dimensions and shifting treatment burdens over time. Longitudinal transition analyses (e.g., Latent Transition Analysis) are urgently needed. Second, the data rely on self-reported instruments (C-eHEALS, PETS), which may be subject to recall bias, although rigorous quality controls were maintained.

Finally, this study employed a convenience sampling strategy based on a single urban tertiary hospital, meaning the sample may not fully represent the broader, highly diverse population of older adults with heart failure across China. This single-center design particularly may limit broad generalizability to highly rural populations where systemic broadband access presents fundamentally different barriers to care. Furthermore, our inclusion criteria requiring basic digital device access inherently introduced selection bias. While methodologically necessary to isolate the second-level digital divide and prevent hardware-induced floor effects, this criterion systematically excluded the most digitally disenfranchised older adults (representing approximately 50% of the demographic based on 2026 national statistics) (19). Consequently, our findings may underestimate the true prevalence of the “Vulnerable” profile in the broader heart failure population, and the external validity of our latent profiles is strictly limited to older adults who have already crossed the first-level digital divide (26, 27).

## 5 Conclusion

In conclusion, this study successfully operationalizes a rigorous, dimension-level latent profile analysis to unmask the profound heterogeneity characterizing older adults managing chronic heart failure. By empirically identifying three distinct latent profiles—Vulnerable, Transitional, and Capable—we highlight that eHealth literacy and treatment burden are inextricably linked at a granular level, heavily governed by advancing age, compounding comorbidity, and social isolation. Recognizing these specific dimensional typologies strongly supports the transition from “one-size-fits-all” digital health deployments in favor of precision care models that deeply respect the varied cognitive capabilities and specific lifestyle burdens of the aging heart failure population.

## Data Availability

The original contributions presented in the study are included in the article/Supplementary material, further inquiries can be directed to the corresponding author.
