# Supplementary material for: Heterogeneity of eHealth literacy and treatment burden in older adults with heart failure: a multidimensional latent profile analysis
Source: Front Public Health. 2026 Jun 2;14:1822855. doi: 10.3389/fpubh.2026.1822855 (PMC13268896; doi:10.3389/fpubh.2026.1822855)
Supplement: Supplementary file 1 [file Table_1.docx]

**Supplementary Table 1. Internal Consistency and Validity Metrics of the C-eHEALS and PETS**

| **Instrument / Domain** | **Number of Items** | **Cronbach's α** | **Construct Validity (CFA Fit Indices)** |
| --- | --- | --- | --- |
| **C-eHEALS (Total)** | 8 | 0.92 | $\chi^{2}/df$=2.41, CFI=0.96, TLI=0.95, RMSEA=0.06 |
| - Information Acquisition | 3 | 0.88 | - |
| - Interactive Evaluation | 4 | 0.85 | - |
| - Decision-Making | 1 | - | - |
| **PETS (Total)** | 32 | 0.88 | $\chi^{2}/df$=2.65, CFI=0.94, TLI=0.93, RMSEA=0.07 |
| - Medical Information | 6 | 0.85 | - |
| - Medication Workload | 2 | 0.82 | - |
| - Medical Appointments | 2 | 0.81 | - |
| - Health Management | 2 | 0.83 | - |
| - Medication Side Effects | 1 | - | - |
| - Diet Burden | 2 | 0.86 | - |
| - Exercise Burden | 2 | 0.84 | - |
| - Medical Expenses | 4 | 0.88 | - |
| - Healthcare System | 2 | 0.82 | - |
| - Social Roles | 4 | 0.87 | - |
| - Physical/Mental Exhaustion | 5 | 0.89 | - |
