# Supplementary material for: Heterogeneity of eHealth literacy and treatment burden in older adults with heart failure: a multidimensional latent profile analysis
Source: Front Public Health. 2026 Jun 2;14:1822855. doi: 10.3389/fpubh.2026.1822855 (PMC13268896; doi:10.3389/fpubh.2026.1822855)
Supplement: Supplementary file 2 [file Table_2.docx]

**Supplementary Table 2. Univariate Multinomial Logistic Regression Analysis of Factors Influencing Latent Profiles**

| **Variables** | **Profile 1: Vulnerable vs. Profile 3: Capable (Ref)** |  | **Profile 2: Transitional vs. Profile 3: Capable (Ref)** |  |
| --- | --- | --- | --- | --- |
|  | **Unadjusted OR (95% CI)** | ***P*-value** | **Unadjusted OR (95% CI)** | ***P*-value** |
| **Age (per 1-year increase)** | 1.21 (1.12, 1.31) | <0.001 | 1.08 (1.01, 1.15) | 0.024 |
| **Education Level** *(Ref: Primary)* |  |  |  |  |
| Secondary | 0.28 (0.14, 0.65) | 0.002 | 0.45 (0.25, 0.82) | 0.009 |
| Tertiary or above | 0.09 (0.03, 0.28) | <0.001 | 0.22 (0.10, 0.48) | <0.001 |
| **Living Arrangement** *(Ref: With family)* |  |  |  |  |
| Living alone | 3.45 (1.60, 7.42) | 0.002 | 1.82 (0.89, 3.75) | 0.105 |
| **NYHA Class** *(Ref: Class I/II)* |  |  |  |  |
| Class III/IV | 4.15 (2.12, 8.15) | <0.001 | 1.95 (1.08, 3.52) | 0.026 |
| **Charlson Comorbidity Index (per 1 unit)** | 2.75 (1.88, 4.02) | <0.001 | 1.62 (1.18, 2.22) | 0.003 |
| **Daily Medications (per 1 medication)** | 1.28 (1.12, 1.48) | <0.001 | 1.12 (0.98, 1.28) | 0.095 |
| *Note:* OR = Odds Ratio; CI = Confidence Interval. The univariate multinomial regression models were estimated independently for each variable without adjusting for other covariates. | | | | |
