# Supplementary material for: Heterogeneity of eHealth literacy and treatment burden in older adults with heart failure: a multidimensional latent profile analysis
Source: Front Public Health. 2026 Jun 2;14:1822855. doi: 10.3389/fpubh.2026.1822855 (PMC13268896; doi:10.3389/fpubh.2026.1822855)
Supplement: Supplementary file 3 [file Table_3.docx]

**Supplementary Table 3. Sensitivity Analysis: Model Fit Indices for Latent Profile Analysis Excluding NYHA Class I Patients (n = 380)**

| **Model** | **AIC** | **BIC** | **aBIC** | **Entropy** | **LMR-LRT (P-value)** | **BLRT (P-value)** | **Class Proportions (%)** |
| --- | --- | --- | --- | --- | --- | --- | --- |
| 1-Class | 7550.10 | 7609.20 | 7561.20 | - | - | - | 100 |
| 2-Class | 6980.40 | 7065.10 | 6996.30 | 0.840 | <0.001 | <0.001 | 36.8 / 63.2 |
| **3-Class** | **6610.15** | **6720.50** | **6630.80** | **0.870** | **0.015** | **<0.001** | **31.1 / 50.0 / 18.9** |
| 4-Class | 6585.30 | 6721.40 | 6610.90 | 0.855 | 0.310 | <0.001 | 5.1 / 26.0 / 50.0 / 18.9 |
| 5-Class | 6555.20 | 6717.00 | 6585.70 | 0.860 | 0.450 | 0.085 | 4.8 / 15.5 / 20.0 / 40.8 / 18.9 |
| *Note:* AIC = Akaike Information Criterion; BIC = Bayesian Information Criterion; aBIC = sample-size adjusted BIC; LMR-LRT = Lo-Mendell-Rubin adjusted Likelihood Ratio Test; BLRT = Bootstrap Likelihood Ratio Test. The sample size for this sensitivity analysis is 380 (excluding 45 asymptomatic patients with NYHA Class I) to rigorously verify the stability of the class structure among symptomatic individuals. Bold formatting indicates the selected optimal model. | | | | | | | |
