# Supplementary material for: Heterogeneity of eHealth literacy and treatment burden in older adults with heart failure: a multidimensional latent profile analysis
Source: Front Public Health. 2026 Jun 2;14:1822855. doi: 10.3389/fpubh.2026.1822855 (PMC13268896; doi:10.3389/fpubh.2026.1822855)
Supplement: Supplementary file 4 [file Image_1.pdf]

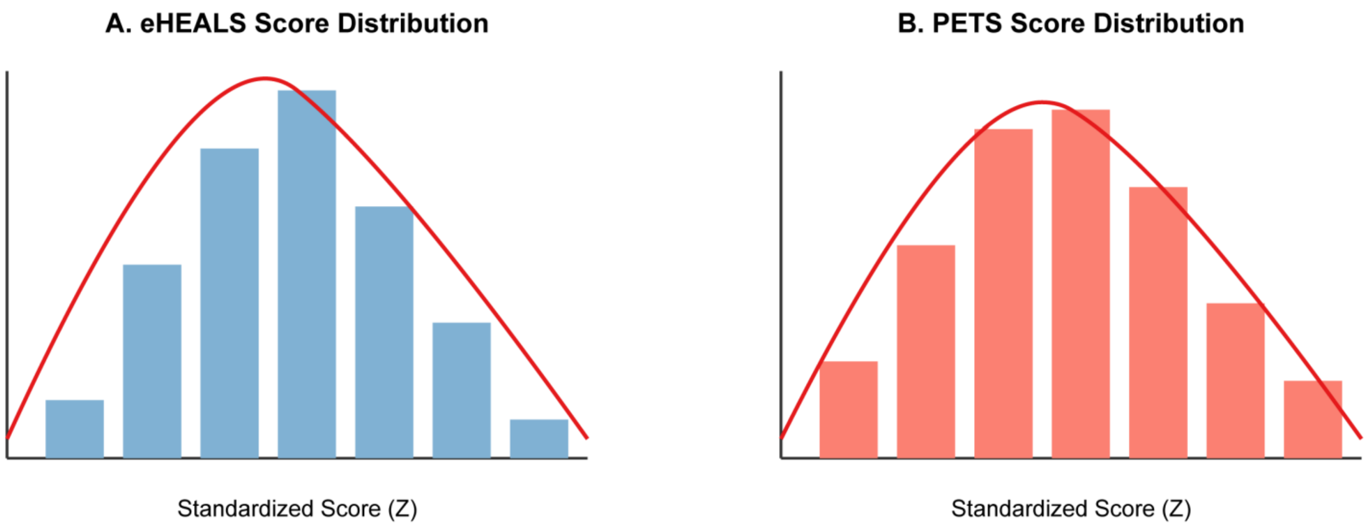


**Supplementary Figure 1. Distribution Histograms of Aggregate Standardized Scores for eHEALS and PETS.** *Abbreviations:* eHEALS, eHealth Literacy Scale; PETS, Patient Experience with Treatment and Self-Management; Z, Standardized Score. *Description:* These two histograms display the overall distribution of the standardized scores (Z-scores) for both the eHEALS (Panel A, blue) and the PETS (Panel B, red) scales across the entire study sample (N = 425) prior to Latent Profile Analysis. The curves approximate a normal distribution, confirming that the continuous variables meet the foundational statistical assumptions necessary for conducting robust Gaussian mixture modeling.
