# Supplementary material for: Heterogeneity of eHealth literacy and treatment burden in older adults with heart failure: a multidimensional latent profile analysis
Source: Front Public Health. 2026 Jun 2;14:1822855. doi: 10.3389/fpubh.2026.1822855 (PMC13268896; doi:10.3389/fpubh.2026.1822855)
Supplement: Supplementary file 5 [file Image_2.pdf]

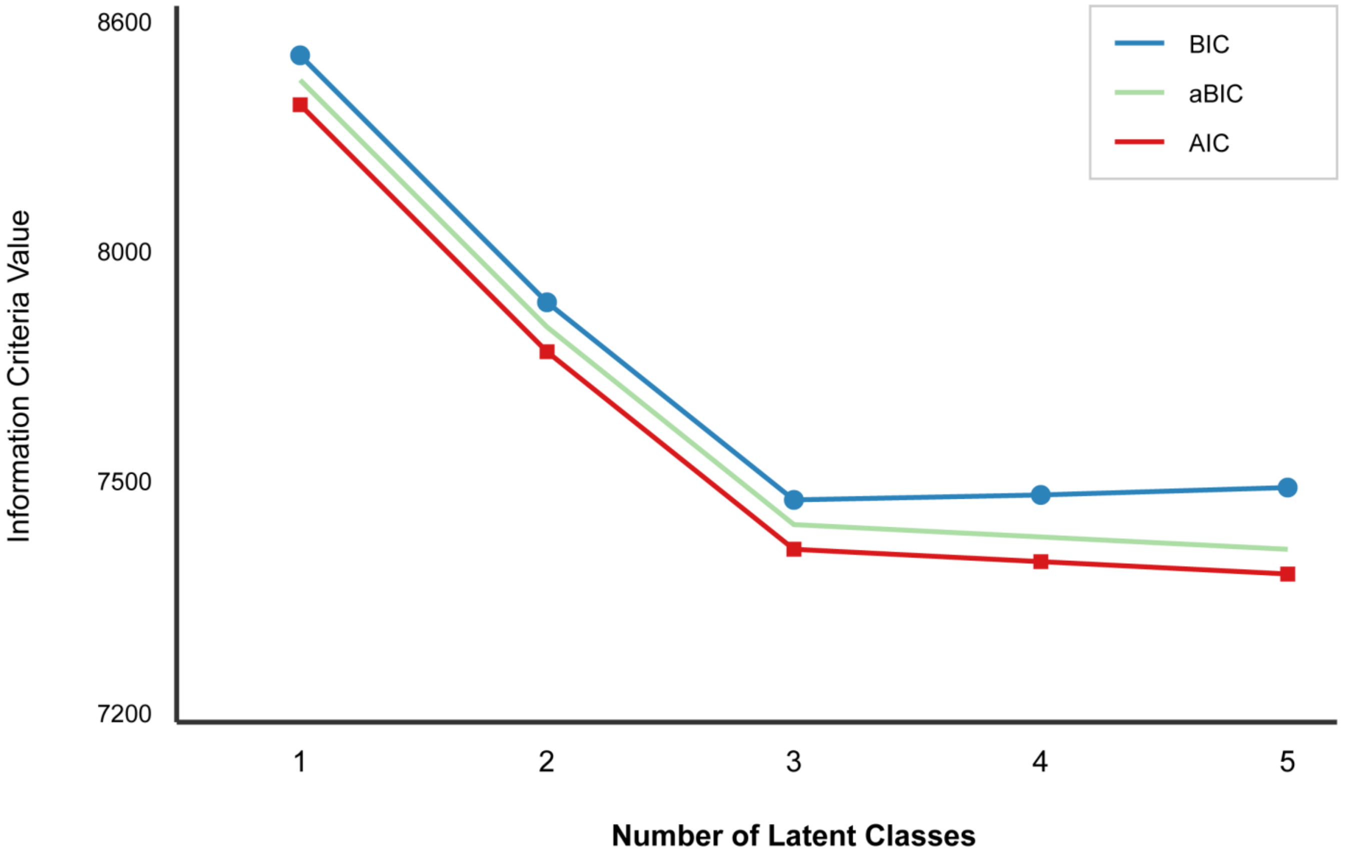

Supplementary Figure 2. Elbow Plot of Information Criteria Across Latent Profile Models. *Abbreviations:* AIC, Akaike Information Criterion; BIC, Bayesian Information Criterion; aBIC, sample-size adjusted Bayesian Information Criterion; LPA, Latent Profile Analysis. *Description:* This elbow plot visualizes the model fit indices (AIC, BIC, and aBIC) for latent profile models specifying 1 to 5 classes. The Y-axis represents the respective information criteria values, while the X-axis indicates the number of latent classes. A distinct inflection point (the "elbow") is observable at the 3-class model, indicating that the extraction of a 3-class solution provides the optimal balance between model fit improvement and statistical parsimony.
